# Unexpected Takotsubo Cardiomyopathy Revealing Postoperative Intra-Abdominal Sepsis: A Case Report

**DOI:** 10.70352/scrj.cr.26-0282

**Published:** 2026-10-01

**Authors:** Takashi Egashira, Toshiaki Nakamura, Takayuki Miyoshi, Haruka Yamasaki, Ken Taniguchi, Yusuke Kajihara, Masayoshi Takeno

**Affiliations:** 1 Department of Intensive Care, Nagasaki Harbor Medical Center, Nagasaki, Nagasaki, Japan; 2 Department of Surgery, Nagasaki Harbor Medical Center, Nagasaki, Nagasaki, Japan; 3 Department of Cardiovascular Medicine, Nagasaki Harbor Medical Center, Nagasaki, Nagasaki, Japan

**Keywords:** Takotsubo cardiomyopathy, postoperative complication, intra-abdominal sepsis, exploratory laparotomy, case report

## Abstract

**INTRODUCTION:**

Takotsubo cardiomyopathy (TTC) is a transient form of left ventricular dysfunction, typically precipitated by intense physical or emotional stress. We report a case in which TTC developed during the postoperative recovery phase and revealed occult intra-abdominal sepsis after surgery for generalized peritonitis.

**CASE PRESENTATION:**

A man in his 60s underwent emergency surgery for generalized peritonitis caused by cecal perforation associated with ascending colon and rectal cancer. Although his postoperative course initially stabilized, diffuse ST-segment elevation and apical hypokinesis developed on POD 5, and TTC was diagnosed. Contrast-enhanced CT showed no definitive evidence of intra-abdominal infection. However, the atypical timing of TTC onset, in conjunction with worsening inflammatory markers and persistently elevated vasopressor requirements, collectively prompted reassessment for an occult surgical complication. Exploratory laparotomy revealed perforation of the rectal stump with an intra-abdominal abscess, and surgical drainage was performed. After reoperation, the patient’s respiratory and circulatory status improved and cardiac function recovered.

**CONCLUSIONS:**

When unexpected TTC develops during postoperative management, the possibility of underlying surgical complications should be considered.

## Abbreviations

ECG
: electrocardiogram
LVEF
: left ventricular ejection fraction
TTC
: Takotsubo cardiomyopathy

## INTRODUCTION

TTC is a reversible form of left ventricular systolic dysfunction that occurs in response to severe psychological or physical stress.^1)^ When TTC occurs during the perioperative period, induction of general anesthesia, cesarean sections, excessive surgical trauma, or pain are considered potential triggers.^2,3)^ However, when TTC develops during the recovery phase, the underlying complications should be suspected.^4)^ We report a case of TTC that developed during the recovery period following surgery for generalized peritonitis. Although definitive imaging findings were lacking, reoperation was performed to investigate the cause of TTC, which ultimately proved life-saving.

## CASE PRESENTATION

A man in his 60s with a history of hypertension managed with antihypertensive medication, and no family history of cardiac disease, developed acute generalized peritonitis caused by cecal perforation associated with ascending colon and rectal cancer. He underwent emergency total colectomy, high anterior resection, and ileostomy, and was subsequently admitted to the ICU. Although a more limited damage-control approach was considered, both the transverse colon and rectum were nearly obstructed by tumors and filled with inspissated feces, posing a high risk of perforation if left in situ. It was therefore judged that resection of the affected segments was necessary for adequate source control, despite the patient’s hemodynamic instability. Despite massive intraoperative fluid resuscitation, his hemodynamic status required high-dose vasopressor support. Broad-spectrum antimicrobial therapy was initiated, along with hydrocortisone (200 mg/day) and polymyxin B–immobilized fiber column hemoperfusion for shock management. Transthoracic echocardiography on admission showed no wall motion abnormalities, and a 12-lead ECG showed no abnormal findings. By POD 3, his blood pressure was maintained with minimal norepinephrine despite a negative fluid balance strategy. The white blood cell count and inflammatory markers showed a downward trend, and the characteristics of the drainage fluid were unremarkable. Surgical drains had been placed in the pouch of Douglas and bilateral subphrenic spaces at the time of initial surgery; however, retrospective review suggested that the drain in the pouch of Douglas was likely partially occluded, which may have impaired adequate drainage of contaminated peritoneal fluid from the pelvic cavity. The patient was weaned from mechanical ventilation on POD 4. The patient’s respiratory status remained stable for several hours after extubation; however, oxygenation gradually deteriorated, necessitating reintubation and mechanical ventilation. Circulatory support required higher doses of norepinephrine and vasopressin than before extubation (**Fig. 1**). On POD 5, monitor electrocardiography showed widespread ST-segment elevation, and a 12-lead ECG confirmed ST-segment elevation in all leads (**Fig. 2**). Transthoracic echocardiography revealed reduced wall motion predominantly in the apical region, with a markedly decreased LVEF of 32%. Coronary angiography showed no significant stenosis, and left ventricular angiography demonstrated basal hyperkinesis with apical akinesis, leading to a diagnosis of TTC (**Fig. 3A** and **3B**). Because the inflammatory markers were elevated and higher vasopressor doses were required, contrast-enhanced CT was performed. However, CT revealed only fluid accumulation consistent with expected postoperative findings. The findings from the surgical drains also provided insufficient evidence to strongly suggest intra-abdominal infection. Therefore, the patient’s respiratory and circulatory instability was initially attributed to TTC. On POD 6, vasopressor requirements remained unchanged, and further increases in the white blood cell count and inflammatory markers were observed. The timing of TTC onset did not correspond to the perioperative course, and the possibility that the fluid collection identified on contrast-enhanced CT represented an abscess could not be excluded. Therefore, an exploratory laparotomy was performed. Upon opening the abdomen, perforation of the rectal stump was identified, and the entire abdominal cavity was contaminated with abscess. The perforation was subsequently closed by suturing after thorough irrigation and drainage. The patient was returned to the ICU under open abdominal management using negative pressure wound therapy (ABThera Open Abdomen Negative Pressure Therapy System; 3M, St. Paul, MN, USA). On POD 8, an additional rectal resection was performed to remove the rectal stump, followed by further irrigation, drainage, and ileostomy creation. The abdomen was then closed. After these curative procedures, the patient’s respiratory and circulatory status gradually improved. He was successfully weaned from mechanical ventilation again on POD 12. Echocardiography on POD 17 demonstrated improvement in apical wall motion and an LVEF of 47%. Vasopressor support was gradually tapered and discontinued. The patient was discharged from the ICU on POD 18. Transthoracic echocardiography at outpatient follow-up confirmed further recovery of cardiac function, with an LVEF of 60%.

**Fig. 1:**
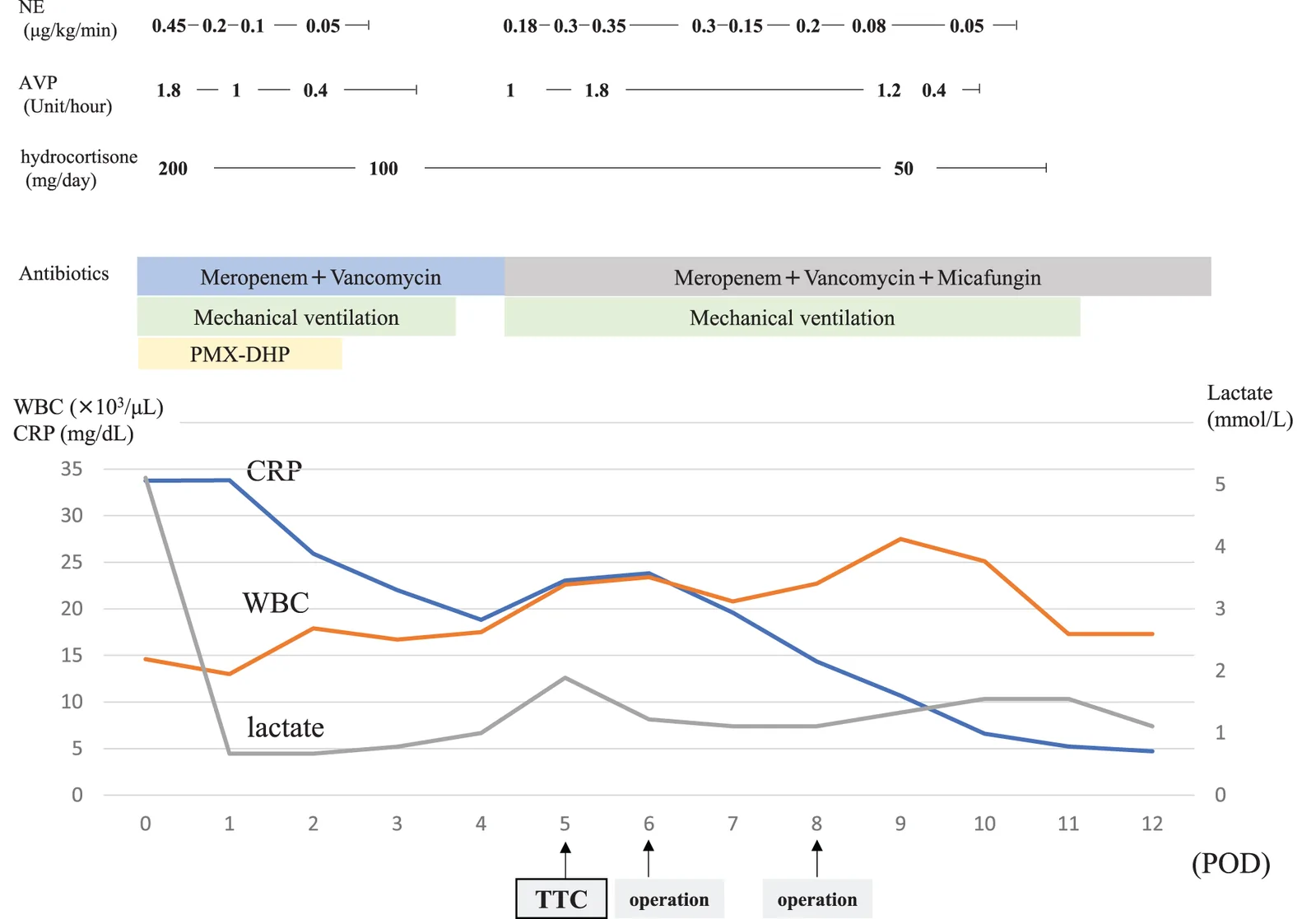
Clinical course during the postoperative period. Vasopressor requirements increased prior to the diagnosis of TTC. AVP, arginine vasopressin; CRP, C-reactive protein; NE, norepinephrine; PMX-DHP, polymyxin B hemoperfusion; POD, POD after the initial surgery; TTC, Takotsubo cardiomyopathy; WBC, white blood cell

**Fig. 2:**
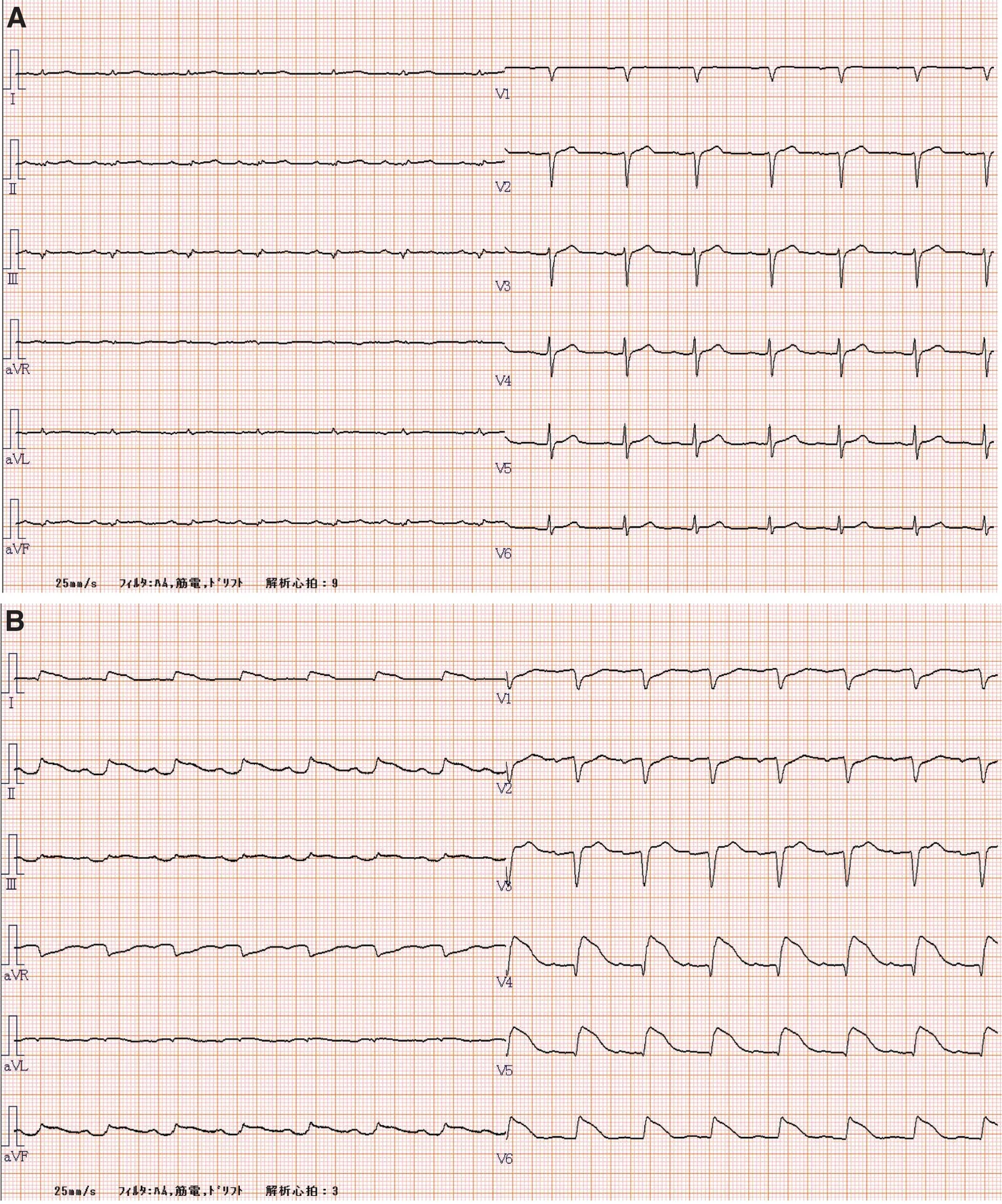
ECG findings. A 12-lead ECG obtained on POD 1 showed normal sinus rhythm without ST-segment changes (**A**). On POD 5, diffuse ST-segment elevation consistent with TCC was observed across all leads (**B**). ECG, electrocardiogram; TTC, Takotsubo cardiomyopathy

**Fig. 3:**
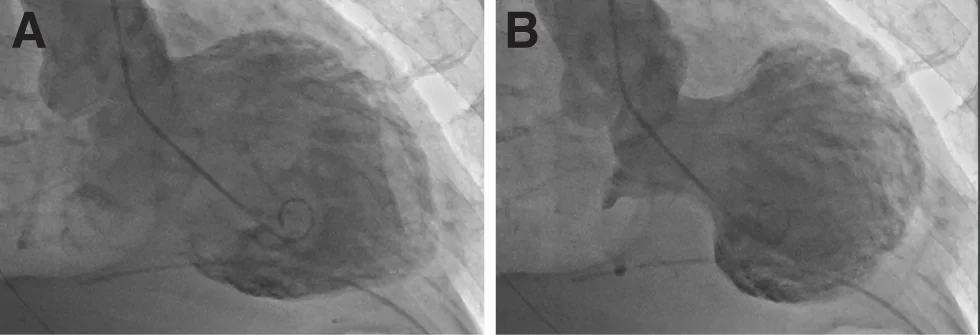
Left ventricular angiography demonstrating apical akinesis with basal hyperkinesis consistent with TTC. (**A**) End-diastolic image showing apical akinesis. (**B**) End-systolic image demonstrating basal hyperkinesis and apical ballooning. TTC, Takotsubo cardiomyopathy

## DISCUSSION

TTC is characterized by transient left ventricular systolic dysfunction with typical apical ballooning in the absence of obstructive coronary artery disease.^5,6)^ The condition is generally triggered by sudden emotional or physical stress and is believed to be mediated by excessive catecholamine release. TTC has increasingly been reported in the perioperative setting. Surgical trauma, anesthesia, postoperative pain, and emotional stress are recognized triggers.^3)^ In addition, sepsis is a known precipitating factor because severe infection can induce marked endogenous catecholamine release and often necessitates exogenous vasopressor administration.^7,8)^ In cases of TTC caused by sepsis, 92.3% of patients have favorable outcomes when appropriate treatment is provided.^9)^ Based on this report, early diagnosis and appropriate treatment are important in TTC associated with postoperative sepsis.

However, most reported cases of perioperative TTC occur immediately after surgery or during the early postoperative period when physiological stress is maximal.^4)^ In contrast, TTC in the present case developed on POD 5 after the patient had temporarily stabilized and been weaned from mechanical ventilation. This timing was atypical for stress related solely to the surgery or anesthesia. Furthermore, although TTC could potentially have been triggered by reintubation or vasopressor use, the catecholamine surge associated with airway manipulation is generally transient.^10)^ In this patient, however, vasopressor requirements progressively increased and inflammatory markers worsened after the onset of TTC, suggesting the presence of an ongoing pathological process rather than transient procedural stress.

Although contrast-enhanced CT did not reveal definitive findings of intra-abdominal infection, the decision to perform re-exploration was ultimately driven by a combination of clinical findings: worsening inflammatory markers, rising white blood cell counts, and persistently elevated vasopressor requirements despite ongoing treatment. In this context, the atypical timing of TTC onset—occurring on POD 5, after an initial period of clinical stabilization—served as an additional warning sign that prompted a more critical reassessment of the patient’s overall trajectory, rather than attributing all deterioration solely to TTC-related cardiac dysfunction. Exploratory laparotomy ultimately revealed rectal stump perforation with diffuse intra-abdominal abscess, confirming uncontrolled intra-abdominal sepsis as the underlying cause.

When TTC develops unexpectedly during postoperative recovery, its onset timing and clinical context should be carefully evaluated rather than regarded in isolation. Clinicians should integrate the onset pattern of TTC with other clinical indicators—including hemodynamic trends, inflammatory markers, and the overall postoperative course—to assess whether an unresolved surgical complication may be contributing to the patient’s condition.

A 2023 systematic review of perioperative TTC in noncardiac surgery, which analyzed 101 cases across published reports, demonstrated that the majority of cases occurred during emergence from anesthesia or on the first POD, when physiological stress is at its peak.^11)^ Cases arising after an initial period of clinical stabilization—as observed in the present case, in which TTC developed on POD 5 following temporary improvement—represent a clinically distinct and less commonly reported pattern. A recent case report described TTC developing 5 days after emergency surgery for sigmoid colon perforation with purulent peritonitis, similarly presenting with markedly reduced LVEF and diffuse ECG changes in the setting of ongoing sepsis.^12)^ However, in that case, the infectious source had been addressed surgically at the initial operation and no reoperation was required. The present case is distinguished by the fact that delayed-onset TTC occurred in the context of an unrecognized ongoing septic focus—rectal stump perforation—that was not identified on CT imaging, and the decision to proceed with exploratory laparotomy was based on a composite of clinical indicators rather than definitive imaging findings. To our knowledge, this specific combination of delayed postoperative TTC onset, inconclusive imaging, and the need for reoperation guided by clinical judgment rather than imaging has not been previously described.

In critically ill patients with sepsis, distinguishing TTC from sepsis-induced cardiomyopathy can be challenging, as both conditions may present with acute left ventricular dysfunction in the absence of significant coronary artery disease. Septic cardiomyopathy is characterized by global myocardial depression, whereas TTC typically demonstrates a regional distribution with characteristic apical ballooning and basal hyperkinesis. In the present case, the diagnosis of TTC was supported by the absence of significant coronary artery disease on angiography and the characteristic apical akinesis with basal hyperkinesis on left ventriculography. Nevertheless, given the patient’s concurrent septic state, a degree of overlap with sepsis-induced myocardial dysfunction cannot be entirely excluded. Regarding hemodynamic management, inotropic agents with beta-adrenergic activity, such as dobutamine, are generally recommended when significant left ventricular systolic dysfunction is present in TTC. In the present case, however, the patient’s hemodynamic status following reoperation was extremely unstable, necessitating a continuous epinephrine infusion for approximately 2 days. Given the concern that additional catecholamine stimulation might exacerbate TTC, dobutamine was avoided; instead, a phosphodiesterase-III inhibitor was administered for inotropic support, while monitoring for signs of end-organ hypoperfusion, including lactate levels and markers of organ dysfunction.

The failure of the surgical drains to detect the intra-abdominal infection in this case merits comment. Drains were placed in the pouch of Douglas and bilateral subphrenic spaces at the initial operation; however, the rectal stump perforation subsequently developed at a site that was not directly accessible to the pelvic drain, and partial drain occlusion is believed to have further limited the drainage of contaminated fluid. This case highlights the limitations of relying solely on drain output for postoperative infection surveillance, particularly following rectal stump closure after high anterior resection. Surgeons should be aware that drain output may remain unremarkable even in the presence of significant occult intra-abdominal contamination when the site of infection is anatomically remote from, or poorly accessed by, the drain.

Reoperation in patients with TTC and reduced cardiac function carries substantial risk.^13)^ However, in the present case, definitive source control through surgical intervention resulted in both hemodynamic stabilization and recovery of cardiac function. Failure to recognize the underlying septic process could have led to progressive septic and cardiogenic shock.

## CONCLUSIONS

When unexpected TTC develops during postoperative management, its onset timing and clinical context should be carefully evaluated. In the present case, the atypical onset of TTC on POD 5, in conjunction with persistently elevated inflammatory markers and increasing vasopressor requirements, collectively prompted reassessment for an occult surgical complication. TTC alone was not the decisive trigger for reoperation; rather, it was 1 of several converging clinical signals that warranted further investigation. This case underscores the importance of integrating unexpected cardiac events into the broader clinical picture during postoperative recovery.

## DECLARATIONS

### Availability of data and materials

All data generated or analyzed during this study are included in this article.
